# The Use of Volatile Irritants in Collapse

**Published:** 1917-10

**Authors:** 


### THE USE OF VOLATILE IRRITANTS IN COLLAPSE

Any one who has followed the results of the modern scientific study of drugs which have long had a place in empiric medicine must have been impressed with many unanticipated disillusionments. The alleged remedial virtues of more than one widely heralded "vegetable remedy" have persistently avoided the inquiring search of the unbiased therapeutic investigator. Alcohol, long vaunted in its various guises as a prince among stimulants, has been forced to accept the demonstrated role of a depressant. Alleged antiseptic washes have proved to retain essentially the hygienic value of cleansing water. The searchlight of truth has no respect for traditions, alone.

These lines are inspired by a timely investigation of the effects of volatile irritants which are among the substances in common use to combat conditions of threatened or actual circulatory failure. It has truly been said that the clinical use of the so-called circulatory stimulants, such as the volatile substances of a highly irritant nature which are given by the subcutaneous or intramuscular injection in shock or allied conditions, is based, not on exact experimental observation, but on casual bedside impression and tradition. It is conceivable that they may act directly on the circulation; or they may provoke a reflex action as a result of the intense irritation of sensory nerve endings which is well known to lead to rise in blood pressure.

At the Department of Pharmacology in the College of Physicians and Surgeons, New York, Lieb and Herrick have watched the effects of injections of alcohol, ether, camphor and ether, camphor and oil, and turpentine in animals decerebrated so that the pain factor would be entirely excluded. When these irritants were introduced directly into the blood stream no direct action on the circulation, other than threatened or actual collapse, could be observed. Injections at various regions of the skin provoked a reflex rise in blood pressure. The sensibility of the sites varied in accord with earlier observations on the comparative sensibility of various parts of the body surface, in the following tested order: (1) the nasal septum, (2) pads of the feet, (3) pads of the hands, and (4) the general body surface. The duration of the response was brief at best, usually less than fifteen minutes, and often so transient that only a few seconds were involved. Any measure which destroyed or profoundly depressed the function of any part of the reflex arc involved prevented any blood pressure raising effect from the subcutaneous injection of the volatile irritants. The investigators, therefore, can not escape the conviction that the transitory rise in pressure which these medicaments produce is entirely reflex in character. The heart plays little or no part in the process, the response being effected through the vasomotor apparatus.

It seems impossible to overlook the bearing of such carefully controlled observations on the use of camphor in oil, alcohol, and allied irritants in clinical routine. They may find a place in cases in which, as the New York pharmacologists express it, with cutaneous sensibility present and reflex irritability preserved, an irritant injection may rouse flagging energies for a momentary emergency. Yet in conditions of abolished reflex irritability, as in anesthesia and shock, the effect is nullified; and in conditions of hemorrhage with falling blood pressure the irritants are of little avail. The possibility of actually inducing shock by the traumatism of the injections should not be lost sight of. For the clinician we must therefore commend a careful consideration of these statements of Lieb and Herrick: In circulatory deficiencies, the result of traumatic or toxic insult to the central nervous system, an irritant injection may fairly be called an added burden and may well accelerate oncoming shock. It is a two-edged sword and may be an instrument of collapse. The use of such a measure to stimulate an anesthetized or profoundly prostrated or unconscious patient has no experimental justification. This places a serious burden of proof on the clinical enthusiast.—Editorial in *Journal A. M. A.*, September 22, 1917.

